# Association of ALI and mCONUT With In‐Hospital De Novo Heart Failure in Patients With STEMI

**DOI:** 10.1002/clc.70526

**Published:** 2026-11-11

**Authors:** Zhiyin Dai, Lijuan Yang, Rui Chen, Ye Zhou, Wei Yuan, Duzhe Jiang, Junjie Liu

**Affiliations:** ^1^ Department of Cardiology Affiliated Hospital of Jiangsu University Zhenjiang Jiangsu China; ^2^ Department of Pediatrics Nanjing Medical University Affiliated Huai'an First Hospital Huai'an Jiangsu China

**Keywords:** advanced lung cancer inflammation index, de novo heart failure, modified controlling nutritional status score, myocardial infarction

## Abstract

**Background:**

De novo heart failure (HF) during the first ST‐segment elevation myocardial infarction (STEMI) hospitalization significantly increases mortality and readmission. While nutritional and inflammatory indicators like the advanced lung cancer inflammation index (ALI) and modified Controlling Nutritional Status (mCONUT) score are gaining attention, their exact association with post‐STEMI de novo HF remains unclear.

**Methods:**

This retrospective study (January 2016 to June 2025) included 1598 first‐time STEMI patients undergoing percutaneous coronary intervention. Patients were categorized into de novo HF and non‐HF groups to evaluate the association of ALI and mCONUT scores with HF development.

**Results:**

The HF group exhibited significantly lower ALI and higher mCONUT scores compared to the non‐HF group. Multivariate logistic regression identified lower ALI and higher mCONUT scores as independent predictors of HF risk. Receiver operating characteristic analysis demonstrated that mCONUT possessed better predictive performance than ALI, with effects remaining highly consistent across subgroups. Sensitivity analyses and NRI/IDI results confirmed that mCONUT provides independent incremental predictive value beyond traditional risk models (optimism‐corrected *C*‐statistic 0.785 vs. 0.751). These associations were independent of admission hemodynamic status (Killip class) and treatment era.

**Conclusion:**

Both ALI and mCONUT scores are independently associated with in‐hospital de novo HF in STEMI patients. The mCONUT score demonstrates superior predictive performance, serving as a promising indicator for early risk stratification.

## Graphical abstract

Among 1598 patients with first‐time STEMI, both the advanced lung cancer inflammation index (ALI) and the modified Controlling Nutritional Status (mCONUT) score independently predicted in‐hospital de novo heart failure. mCONUT outperformed ALI (AUC 0.706 vs. 0.611) and added significant incremental prognostic value, supporting early nutritional inflammatory risk stratification.

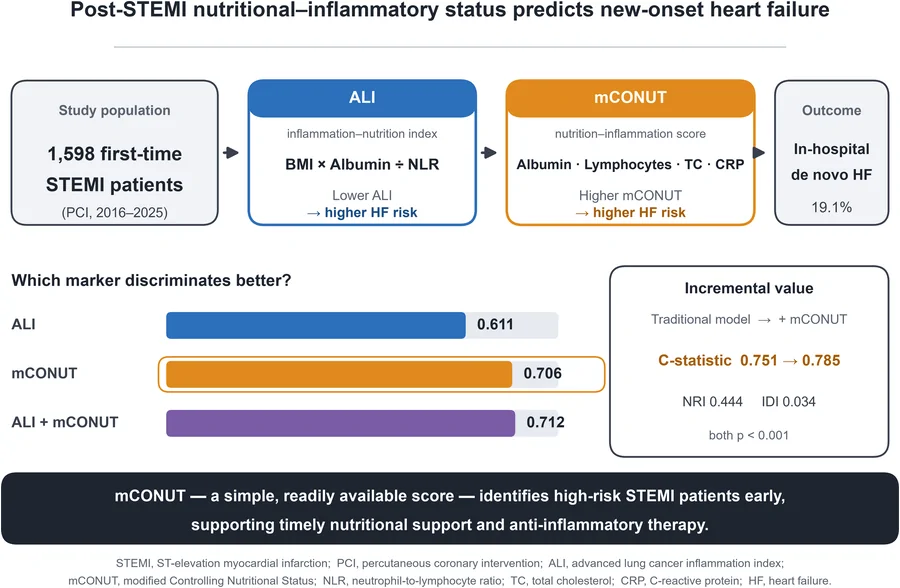

## 1 Introduction

De novo heart failure (HF) during hospitalization for acute myocardial infarction (AMI) significantly increases short‐ and long‐term mortality, increases the risk of readmission and adverse cardiovascular events, and severely reduces quality of life [1]. Traditional risk factors for AMI combined with HF include underlying heart disease, large myocardial infarction, delayed reperfusion therapy, advanced age, hypertension, diabetes, and abnormal biomarkers such as brain natriuretic peptide (BNP) [2]. Nontraditional risk factors including nutritional and metabolic abnormalities [3], abnormal inflammatory and immune markers [4], frailty, and psychological and social factors, have gained increasing attention as potential influencing factors in recent years, providing new insights into the early identification and risk stratification of de novo HF in patients with AMI.

Myocardial infarction‐induced local hypoxia leads to cardiomyocyte necrosis, which in turn activates the immune system to clear debris and initiate reparative scar formation [5]. However, severe tissue damage can induce an uncontrolled inflammatory cascade: early on, neutrophils produce large amounts of reactive oxygen species, release granzymes, and neutrophil extracellular traps; subsequently, macrophages are overactivated and secrete pro‐inflammatory cytokines, further exacerbating the spread of inflammation and the expansion of the infarct area [6]. This excessive inflammatory response not only leads to pathological fibrosis and a persistent autoimmune response but also drives adverse cardiac remodeling, eventually progressing to HF [7]. Simultaneously, malnutrition can impair the body's repair capacity and delay cardiac recovery. Ultimately, excessive inflammation and malnutrition often form a vicious cycle, exacerbating the complexity of AMI and significantly increasing short‐term mortality and complication rates [8]. Despite this understanding, routine clinical risk stratification often relies on hemodynamic and demographic variables [9], potentially overlooking this specific inflammatory‐metabolic substrate.

To address this, composite indices integrating nutritional and inflammatory markers have gained attention. Studies have shown that composite indices such as the monocyte‐to‐high density lipoprotein cholesterol ratio [10] and high triglyceride glucose index levels [11] are associated with the severity of AMI and higher mortality. The advanced lung cancer inflammation index (ALI) is a composite indicator comprising body mass index (BMI), serum albumin, and the neutrophil‐to‐lymphocyte ratio (NLR), serves as a proxy for the balance between nutritional reserve and systemic inflammation [12]. The modified Controlling Nutritional Status (mCONUT) score, which is based on the original CONUT, comprises a weighted score consisting of four components: albumin, lymphocyte count, total cholesterol (TC), and C‐reactive protein (CRP) [13]. Conceptually, while ALI emphasizes the cellular inflammatory balance (NLR) and physical reserve (BMI), mCONUT provides a broader assessment of the immune‐metabolic axis, capturing acute‐phase reactants and lipid metabolism, which are crucial in the post‐infarct metabolic shift. Unlike traditional risk scores such as the Global Registry of Acute Coronary Events score or Killip classification, which primarily reflect clinical severity and hemodynamic instability at presentation [14], ALI and mCONUT quantify the underlying biological milieu of inflammation and malnutrition. Previous studies have independently linked low ALI to poor left ventricular reverse remodeling [15] and high CONUT scores to major adverse cardiovascular events in ST‐segment elevation myocardial infarction (STEMI) patients [16]. However, the incremental value of such biomarkers over traditional models remains underexplored, and direct comparative studies between the two in predicting new‐onset HF acutely driven by inflammatory storms have yet to be elucidated. Therefore, this study aimed to assess the association and prognostic value of ALI and mCONUT in STEMI patients with de novo HF.

## 2 Methods

### 2.1 Design and Study Population

This retrospective study enrolled consecutive adult patients (aged ≥ 18 years) diagnosed with a first STEMI who underwent PCI at the Affiliated Hospital of Jiangsu University between January 2016 and June 2025. Diagnosis and treatment were conducted in accordance with the 2015 Guidelines for the Diagnosis and Treatment of Acute ST‐segment Elevation Myocardial Infarction in China [17]. Patients with (1) documented HF, (2) history of AMI or prior coronary revascularization, including PCI or coronary artery bypass grafting (CABG), (3) malignant tumor, (4) acute infection, (5) autoimmune disease, (6) severe hepatic or renal insufficiency, (7) missing baseline data, or (8) who declined coronary angiography or primary PCI were excluded. Excluding these patients aimed to eliminate confounding factors that could independently influence inflammation‐nutritional status or the risk of HF, ensuring that the study cohort accurately reflects de novo HF after the first STEMI without serious underlying diseases or competing risks. All patients who met the inclusion criteria and did not meet any exclusion criteria were enrolled in the study. The study initially involved 1728 STEMI patients. After excluding 39 patients with a history of PCI or CABG, 1689 patients with first‐time STEMI were identified. Following further exclusion of 91 patients due to missing baseline data (*n* = 24), prior HF (*n* = 8), acute infection (*n* = 28), autoimmune disease (*n* = 5), malignant tumor (*n* = 8), severe liver or kidney damage (*n* = 13), and rejected coronary angiography or primary PCI (*n* = 5), a final cohort of 1598 patients was included for analysis (Figure 1). This study was conducted and reported in accordance with the REporting of studies Conducted using Observational Routinely‐collected health Data guidelines, with reference to the corresponding checklist.

**Figure 1:**
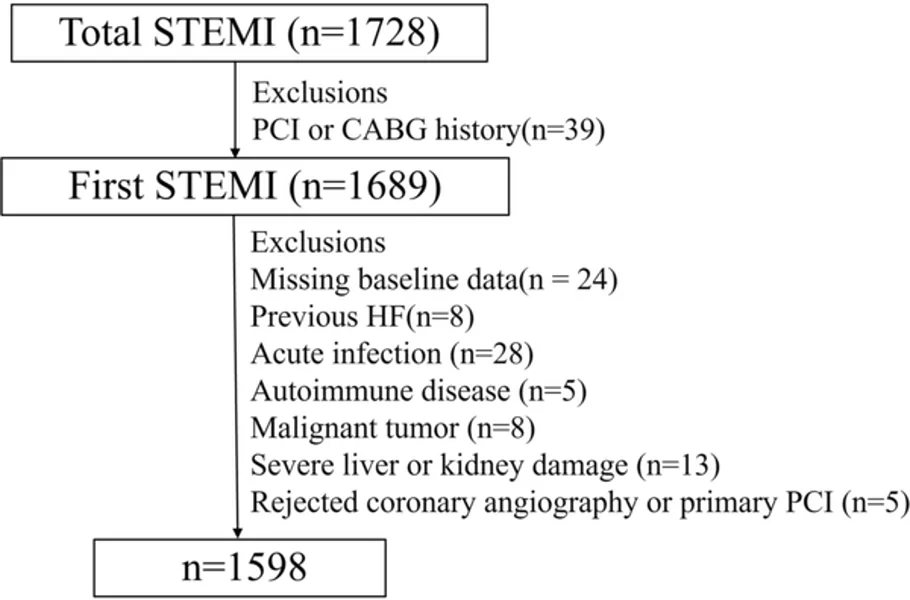
Flow diagram of study population enrollment. CABG, coronary artery bypass grafting; HF, heart failure; PCI, percutaneous coronary intervention; STEMI, ST‐segment elevation myocardial infarction.

### 2.2 Data Acquisition and Criteria

Trained and blinded physicians collected clinical data from medical records. These included demographic characteristics, medical history (hypertension, diabetes, smoking), vital signs (systolic blood pressure [SBP], diastolic blood pressure [DBP], heart rate), and laboratory and imaging parameters. BMI was calculated as weight (kg)/height (m^2^); hypertension was defined by prior diagnosis, medication use, or blood pressure ≥ 140/90 mmHg on three different days post‐admission.

Diabetes was diagnosed per the 2020 Chinese guidelines for type 2 diabetes [18], based on standard glycemic criteria. All blood pressure and heart rate measurements were taken upon hospital admission; blood samples for markers were collected fasting on the morning following admission, representing the baseline status prior to the potential development of in‐hospital HF. To ensure data consistency across the study period, all laboratory assays were performed using standardized automated analyzers calibrated annually according to manufacturer specifications. If multiple echocardiograms were performed, the lowest left ventricular ejection fraction (LVEF) value was recorded; the peak values of BNP and troponin were calculated. According to the 1997 World Health Organization criteria, smoking was determined by a lifetime of 6 months or more of continuous or cumulative smoking [19]. ALI was calculated as (BMI × serum albumin [g/dL])/neutrophil‐to‐lymphocyte ratio. The CONUT score includes three indicators: albumin level, lymphocyte count, and TC. Based on references, a modified CONUT score was developed by incorporating CRP levels, a new inflammatory marker, into the original CONUT score. The mCONUT score was developed by Zhang et al. [13] by incorporating CRP into the original CONUT scoring system. It comprises four parameters: serum albumin (≥ 35 g/L = 0 points; 30–34.9 = 2; 25–29.9 = 4; < 25 = 6), peripheral lymphocyte count (≥ 1.6 × 10^9^/L = 0; 1.2–1.59 = 1; 0.8–1.19 = 2; < 0.8 = 3), TC (≥ 4.65 mmol/L = 0; 3.62–4.64 = 1; 2.58–3.61 = 2; < 2.58 = 3), and CRP, which is double‐weighted (≤ 5 mg/L = 0; 5.01–10 = 2; 10.01–35 = 4; > 35 = 6). CRP concentrations were measured and expressed in mg/L. Values exactly located at a cut‐off were assigned to the lower‐score category. The total mCONUT score therefore ranges from 0 to 18 points, with higher scores indicating a worse nutrition–inflammation status; the detailed scoring criteria are presented in Supporting Information S1: Table S1.

### 2.3 Coronary Angiography

The cardiologists comprehensively determined whether an emergency cardiac catheterization was necessary based on the patient's clinical condition, including the presence of persistent chest pain, electrocardiographic findings suggestive of STEMI, and the presence of cardiogenic shock. Coronary angiography is performed in strict accordance with standard operating procedures and technical specifications. Two independent cardiologists jointly review and evaluate the angiographic images. Significant coronary stenosis (≥ 50% luminal narrowing in any major artery) was defined per angiographic criteria. The infarct‐related artery was specified as the vessel with the most severe stenosis. Symptom onset‐to‐balloon (So‐to‐B) time was measured from chest pain onset to successful revascularization.

### 2.4 Outcomes

The endpoint is now defined as de novo HF strictly assessed and confirmed during the index hospitalization, adjudicated by two independent cardiologists blinded to ALI/mCONUT values, with a third senior cardiologist resolving any discrepancies. Patients met the endpoint if they developed dyspnea plus at least one objective sign of congestion (pulmonary rales, lower‐extremity edema, pulmonary congestion on chest X‐ray, third heart sound with sustained tachycardia, or need for intravenous diuretics/vasodilators). Presentation Killip class ≥II, pulmonary edema, and cardiogenic shock were recorded as additional supportive evidence. The time to HF onset was calculated as the interval between hospital admission and the clinical occurrence of de novo HF. We also justify the NT‐proBNP < 300 pg/mL (or BNP < 100 pg/mL) exclusion thresholds as consistent with acute HF rule‐in/rule‐out limits in current European Society of Cardiology guidelines.

### 2.5 Statistics

All analyses were performed using Python (version 3.13). Numerical variables were first tested for normal distribution. For non‐normally distributed numerical data, results are presented as median (interquartile range), and the Mann−Whitney *U* test was used for two‐group comparisons. For quantitative data with three or more non‐normally distributed groups, the Kruskal−Wallis *H* test was used. Categorical variables are presented as numbers (percentages), and comparisons between groups were made using the chi‐square or Fisher's exact test. Correlations between ALI, mCONUT, and clinical data or laboratory test results were analyzed using Spearman's rank correlation test. Univariate logistic regression was used for preliminary analyses. Significant variables were selected using *p* < 0.05 as the criterion and then included in the multivariate model for adjustment. Events per variable (EPV) was used to evaluate whether the logistic regression models were overfitted. Multicollinearity among independent variables was assessed using the variance inflation factor (VIF), with a cutoff value of < 5 for inclusion in the model. Receiver operating characteristic (ROC) curves were used to assess the predictive value of BMI, albumin, CRP, NLR, TC, ALI, and mCONUT for de novo HF. Cutoff values and diagnostic accuracy for ALI and mCONUT were determined. DeLong's test was used to compare differences in area under the curve (AUC) between BMI, albumin, CRP, NLR, TC, ALI, and mCONUT. Calibration curves were plotted to assess the consistency between measured outcomes and predicted probabilities. The Hosmer−Lemeshow test was used to evaluate the overall goodness of fit. The Net Reclassification Index (NRI) and Integrated Discriminant Improvement Index (IDI) were used to evaluate the incremental discriminant ability of ALI and mCONUT to detect the occurrence of HF based on a traditional model comprising age, sex, history of diabetes, smoking history, reperfusion time, uric acid, DBP, and heart rate. The subgroup analysis in this study was conducted as follows: STEMI patients were stratified by gender, age, smoking history, history of hypertension, and history of diabetes. Within each subgroup, adjusted ORs with 95% confidence intervals (CIs) were calculated for the association of ALI and mCONUT with the study endpoint. Given that the proportion of missing data was low (< 5% for all variables; specifically 4.1% for ALI and 0% for mCONUT) and assumed to be missing completely at random, we employed complete case analysis for the primary models. This method minimizes potential bias associated with data imputation in scenarios with negligible missingness. Differences in associations across subgroups were tested using interaction *p* values, and the results were presented in a forest plot. A two‐sided *p* < 0.05 was considered statistically significant. Length of hospital stay was excluded from all multivariable models because it is strongly influenced by the occurrence of HF, and its inclusion would introduce overadjustment and reverse causation. To strengthen the assessment of incremental value, optimism‐corrected performance was estimated using bootstrap internal validation (400 resamples) for the *C*‐statistic, calibration‐in‐the‐large, calibration slope, and Brier score; decision curve analysis was performed to evaluate clinical utility across threshold probabilities; and continuous (category‐free) NRI and IDI were estimated with bootstrap 95% CIs. Additional sensitivity analyses were performed as follows: (i) adjustment for treatment era, approximated by the hospital admission serial number (a monotonically increasing calendar‐time proxy) and modeled both continuously and in quartiles; (ii) temporal split‐sample validation, in which the model was developed in the early cohort and evaluated in the independent later cohort; (iii) analyses additionally adjusting for the Killip class or restricted to patients with Killip class I at admission; and (iv) alternative assignments of borderline mCONUT values and alternative CRP cut‐off schemes.

## 3 Results

### 3.1 Comparison of Clinical Characteristics of HF and Non‐HF STEMI Patients

Table 1 compares the clinical characteristics of STEMI patients with (*n* = 306) and without (*n* = 1292) de novo HF. HF occurred in 19.1% of all STEMI patients during hospitalization. Compared to the non‐HF group, patients with HF were significantly older, less often male, and had a higher prevalence of diabetes (all *p* < 0.001). The HF group also had significantly higher BNP levels, troponin levels, heart rates, and longer hospital stays (all *p* < 0.001). Furthermore, their LVEF and serum albumin levels were significantly lower than those observed in non‐HF patients (all *p* < 0.001). Patients with HF also demonstrated significantly higher levels of serum creatinine, uric acid, and CRP and lower levels of triglycerides and TC. No significant discrepancies were found across the two groups regarding hypertension, aspartate aminotransferase (AST), and high‐density lipoprotein cholesterol. Overall, patients with de novo HF after STEMI exhibited more severe disease and worse metabolic markers.

**Table 1:** Baseline characteristics according to de novo heart failure.

	Heart failure (*N* = 306)	Non‐heart failure (*N* = 1292)	*p* value
Age (years)	71 (62, 79)	62 (53, 71)	< 0.001
Male sex, %	67.6	82.8	< 0.001
Current smoker, %	40.5	49.1	0.002
DM, %	39.9	27.6	< 0.001
HTN, %	63.4	61.5	0.085
BMI (kg/m^2^)	23.8 (21.2, 25.9)	24.44 (22.4, 26.6)	< 0.001
BNP (pg/mL)	1470.0 (1127.5, 2050.0)	219.5 (92.0, 432.5)	< 0.001
TNI (ng/mL)	25.1 (11.1, 30.0)	19.5 (8.0, 30.0)	< 0.001
LVEF (%)	47 (39, 58)	61 (56, 65)	< 0.001
LOS (days)	12.0 (10.0, 16.0)	10.0 (9.0, 12.0)	< 0.001
ALT (U/L)	50.8 (27.7, 94.9)	45.4 (28.9, 68.2)	0.004
AST (U/L)	167.0 (60.9, 392.7)	156.8 (78.1, 277.4)	0.125
Albumin (g/L)	35.1 (32.6, 37.6)	37.4 (35.4, 39.4)	< 0.001
Creatinine (μmol/L)	85.5 (67.7, 109.3)	70.0 (61.1, 82.2)	< 0.001
Uric acid (μmol/L)	363.0 (288.8, 451.3)	325.0 (267.0, 386.0)	< 0.001
FBG (mmol/L)	7.7 (5.79, 11.42)	6.1 (5.24, 8.21)	< 0.001
TG (mmol/L)	1.3 (0.9, 1.9)	1.8 (1.2, 2.7)	< 0.001
TC (mmol/L)	4.4 (3.6, 5.1)	4.6 (3.8, 5.3)	0.001
HDL‐C (mmol/L)	1.0 (0.8, 1.2)	0.9 (0.8, 1.1)	0.292
LDL‐C (mmol/L)	2.6 (2.0, 3.2)	2.7 (2.2, 3.3)	0.002
CRP	6.6 (1.5, 30.8)	2 (0.5, 5.4)	< 0.001
SBP (mmHg)	120 (106, 133)	123 (110, 136)	0.032
DBP (mmHg)	73 (65, 84)	75 (67, 85)	0.010
HR (bpm)	86 (75, 99)	80 (70, 90)	< 0.001
So‐to‐B (hours)	6.0 (3.0, 16.8)	4.4 (3.0, 9.2)	< 0.001

Abbreviations: ALT, alanine aminotransferase; AST, aspartate aminotransferase; BMI, body mass index; BNP, b‐type natriuretic peptide; CRP, C‐reactive protein; DBP, diastolic blood pressure; DM, diabetes mellitus; FBG, fasting blood sugar; HDL‐C, high density lipoprotein cholesterol; HR, heart rate; HTN, hypertension; LDL‐C, low density lipoprotein cholesterol; LOS, length of stay; LVEF, left ventricular ejection fraction; SBP, systolic blood pressure; So‐to‐B, symptom onset‐to‐balloon; TC, total cholesterol; TG, triglycerides; TNI, troponin I.

### 3.2 Differences in ALI and mCONUT Between the Non‐HF and HF Groups

As Figure 2A illustrates, ALI values were significantly higher in the non‐HF group than in the HF group (14.35 [8.92, 24.86] vs*.* 11.10 [6.52, 17.26], *p* < 0.001). As shown in Figure 2B, the mCONUT values were significantly higher in the HF group than in the non‐HF group (5 [3, 8] vs. 3 [1, 5], *p* < 0.001). Using the mCONUT score to assess nutritional status, the HF group had a markedly higher prevalence of moderate to severe malnutrition (mCONUT score > 7) than the non‐HF group (39.5% vs. 12.9%, *p* < 0.001).

**Figure 2:**
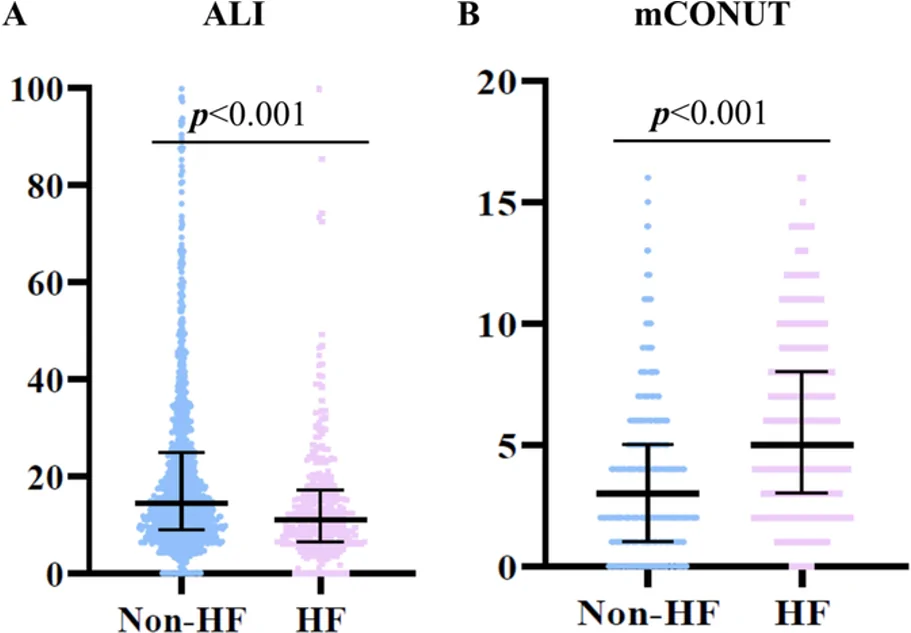
ALI (A) and mCONUT (B) levels distribution at admission in the HF and non‐HF group. ALI, advanced lung cancer inflammation index; HF, heart failure; mCONUT, modified Controlling Nutritional Status.

### 3.3 Correlates of ALI, mCONUT

Spearman's correlation analysis revealed that ALI was negatively correlated with age, BNP, troponin, ALT, AST, Cr, and heart rate (all *p* < 0.001), and positively correlated with LVEF (*p* = 0.008), triglycerides, SBP, and DBP (all *p* < 0.001). Conversely, mCONUT was negatively correlated with LVEF, ALT, AST, triglycerides, SBP, and DBP (all *p* < 0.05), and positively correlated with age, troponin, BNP, Cr, heart rate, So‐to‐B, and CRP (all *p* < 0.05). For detailed correlation coefficients (*r*), see Supporting Information S1: Figure S1.

### 3.4 Correlation Between ALI, mCONUT, and De Novo HF in STEMI

We first performed a univariate logistic regression analysis, revealing that the following were risk factors for de novo HF after STEMI: BNP, troponin I, LVEF, length of hospital stay, ALT, AST, albumin, creatinine, uric acid, fasting blood glucose, triglycerides, TC, LDL‐C, DBP, heart rate, history of diabetes, smoking, reperfusion time, age, sex, BMI, CRP, ALI, and mCONUT (all *p* < 0.05, see Supporting Information S1: Table S2). Except for ALT and AST, whose VIFs were 5.6 and 5.9, respectively, the VIFs of all other variables ranged from 1.09 to 2.14, indicating that ALT and AST were collinear with ALI and mCONUT scores. Thus, ALT and AST were excluded from the adjusted model. Furthermore, as ALI is directly derived from BMI and albumin, BMI and albumin were not adjusted for as confounders in the model. mCONUT is derived from albumin, TC, and CRP; thus, albumin, TC, and CRP were not adjusted for as confounding factors in the model. The EPV in the fully corrected model is 19.1. Both unadjusted and adjusted models showed significant associations: lower ALI with elevated HF risk, and higher mCONUT scores with increased risk (Figure 3). In the fully adjusted models (from which length of hospital stay was excluded), the ORs were 0.999 (95% CI: 0.998−1.000, *p* = 0.003) for ALI and 1.174 (95% CI: 1.121−1.230, *p* < 0.001) for mCONUT. Given that a single‐unit change of ALI may not reflect typical physiological variations, we additionally reported the associations per standard deviation increment. For every 1 standard deviation increase in ALI, the risk of HF decreased by approximately 3.0%.

**Figure 3:**
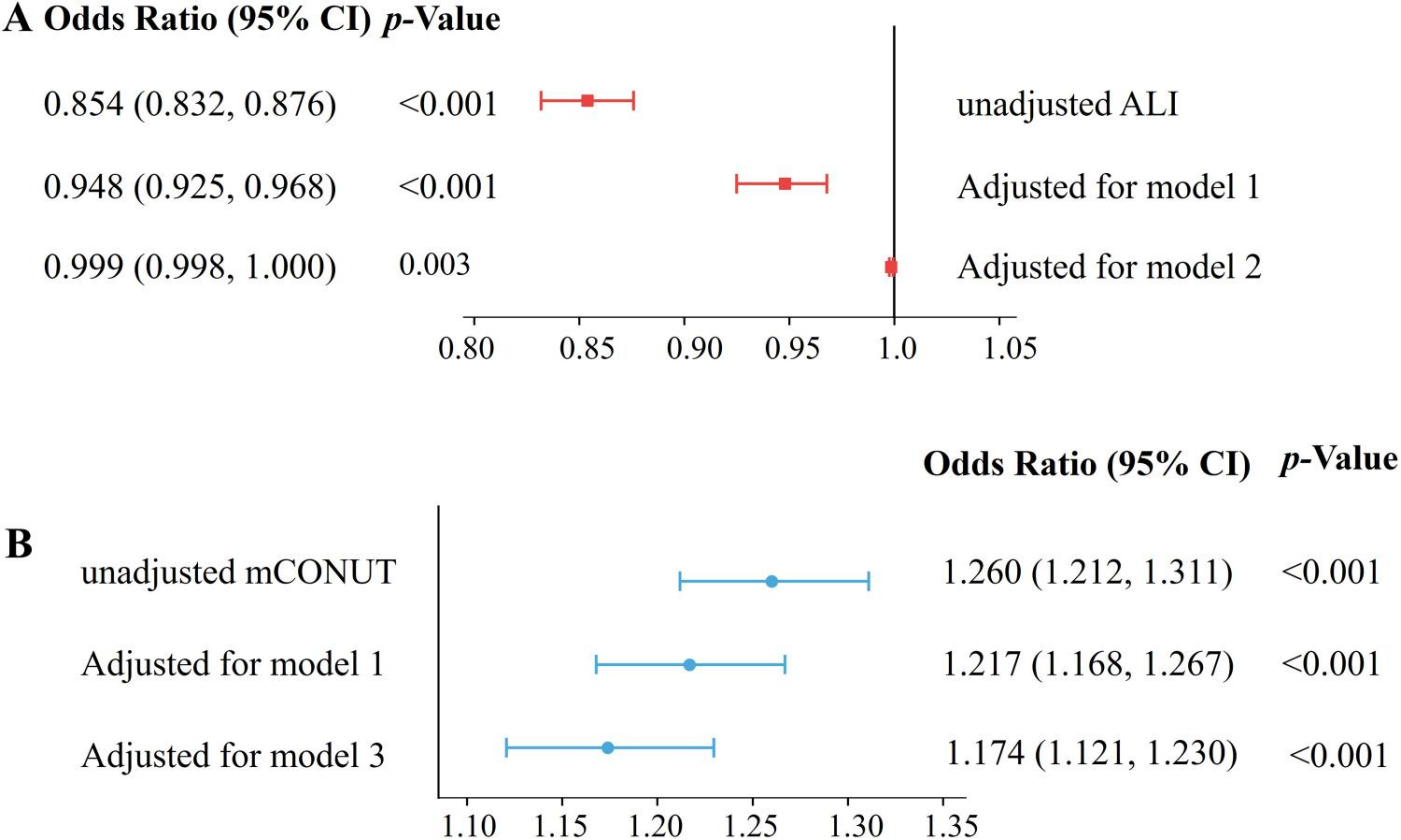
Association between ALI (A)/mCONUT (B) and incidence of de novo heart failure. Model 1: Adjusted for age and sex. Model 2: Adjusted for BNP, troponin I, LVEF, creatinine, uric acid, CRP, fasting blood glucose, triglyceride, diastolic blood pressure, heart rate, history of diabetes, smoking history, reperfusion time, age, and sex. Model 3: Adjusted for BNP, troponin I, LVEF, creatinine, uric acid, fasting blood glucose, triglyceride, diastolic blood pressure, heart rate, history of diabetes, smoking history, reperfusion time, age, sex, and BMI. ALI, advanced lung cancer inflammation index; HF, heart failure; mCONUT, modified Controlling Nutritional Status; OR, odds ratio; 95% CI, 95% confidence interval.

### 3.5 Differences in HF Incidence Across ALI and mCONUT Level Groups

ALI values were ranked from smallest to largest (Figure 4) and then divided into four equal quartiles, designated Q1−Q4. mCONUT scores were divided into Q1−Q4 according to reference values. As shown in Figure 4A, as the ALI quartiles increased from Q1 to Q4, the HF incidence trended downward, and the difference was significant (*p* < 0.001). As shown in Figure 4B, as the mCONUT quartiles increased from Q1 to Q4, the HF incidence trended upward, and the difference was also significant (*p* < 0.001).

**Figure 4:**
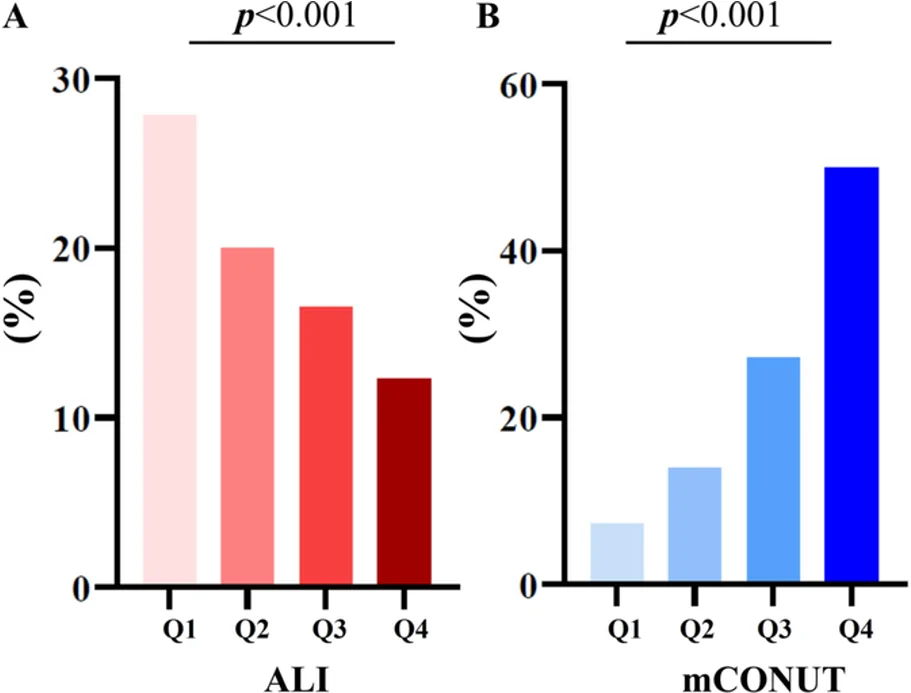
Distribution of heart failure across quartiles of ALI (A) and mCONUT (B). ALI, advanced lung cancer inflammation index; mCONUT, modified Controlling Nutritional Status.

### 3.6 Analysis of the Predictive Value of ALI and mCONUT Scores for HF Development

ROC curve analysis showed that ALI had some discriminatory ability for in‐hospital HF in STEMI patients, with an AUC of 0.611 (95% CI: 0.575–0.646, *p* < 0.001) (Figure 5A). Based on the principle of maximizing the Youden index (Youden index = 0.164), the optimal cutoff value for ALI was 17.239 (sensitivity: 75.3%, specificity: 41.1%). In contrast, mCONUT showed moderate discriminative ability, with an AUC of 0.706 (95% CI: 0.674–0.739, *p* < 0.001) (Figure 5B); its optimal cutoff value (Youden index = 0.411) was 4.5 (sensitivity: 67.3%, specificity: 73.8%).

**Figure 5:**
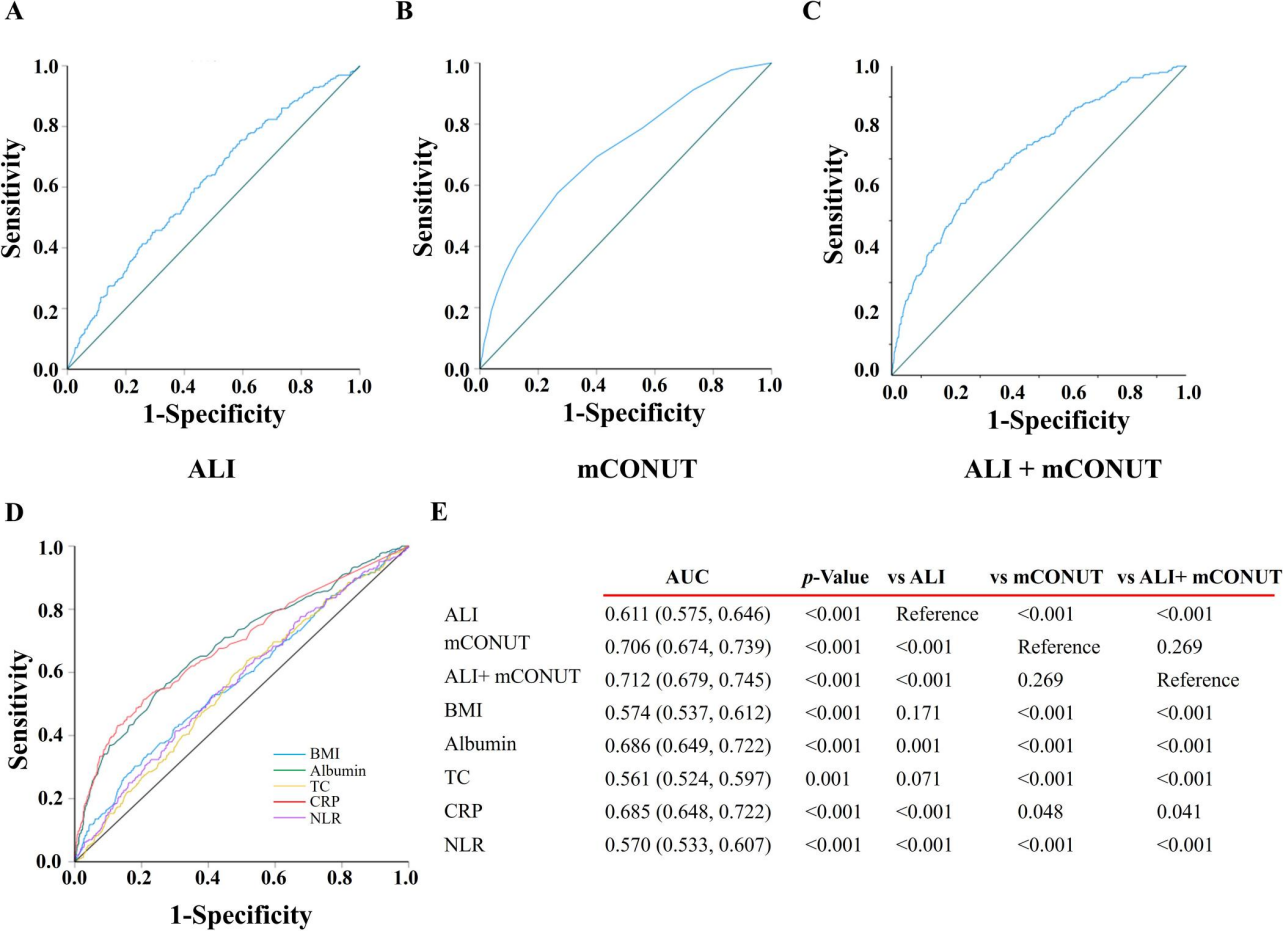
Receiver operating characteristic (ROC) curves for the prediction of de novo heart failure in patients with STEMI. (A) The ROC curve for ALI alone showed an AUC of 0.611. (B) The ROC curve for mCONUT alone showed an AUC of 0.706. (C) The ROC curve for the combined ALI and mCONUT model showed an increased AUC of 0.712. (D) The AUCs for traditional indicators such as BMI, albumin, TC, CRP, and NLR were also compared. (E) Statistical comparisons confirmed that the AUCs of both the mCONUT and ALI+mCONUT combined models were significantly higher than those of ALI and other traditional clinical indicators (all *p* < 0.05), and there was no significant difference between the combined model and mCONUT alone. ALI, advanced lung cancer inflammation index; AUC, area under the curve; BMI, body mass index; CRP, C‐reactive protein; mCONUT, modified Controlling Nutritional Status; NLR, neutrophil‐to‐lymphocyte ratio; TC, total cholesterol.

Although the AUC of the combined ALI and mCONUT model slightly increased to 0.712 (95% CI: 0.679–0.745, *p* < 0.001) (Figure 5C), the DeLong test showed no statistically significant difference in AUC between the combined model and mCONUT alone (*p* = 0.269), suggesting that mCONUT is the main driver of predictive performance. As shown in Figure 5D,E, in comparisons with traditional indicators, mCONUT showed significantly better discriminative power than BMI, TC, and NLR; although the AUCs of albumin and CRP were higher than ALI, they were still lower than mCONUT and its combined model. Calibration curves (Supporting Information S1: Figure S2) and the Hosmer−Lemeshow test (ALI: *p* = 0.108; mCONUT: *p* = 0.279) confirmed the good fit of the model. It is noteworthy that although mCONUT's absolute discriminative power is moderate, adding mCONUT to the traditional model yielded a statistically significant incremental value: the continuous NRI was 0.444 (95% CI 0.313–0.566, *p* < 0.001) and the IDI was 0.034 (95% CI 0.021–0.047, *p* < 0.001) (Table 2). In contrast, adding ALI produced a substantially smaller increment (continuous NRI 0.300; IDI 0.009).

**Table 2:** NRI and IDI analysis for the addition of ALI and mCONUT to the traditional model.

	NRI		IDI	
	Value (95% CI)	*p* value	Value (95% CI)	*p* value
Traditional model	Reference		Reference	
Traditional model + ALI	0.300 (0.158−0.431)	0.001	0.009 (0.001−0.028)	0.009
Traditional model + mCONUT	0.444 (0.313−0.566)	< 0.001	0.034 (0.021−0.047)	< 0.001

*Note:* Traditional model: age, sex, history of diabetes, smoking history, reperfusion time, uric acid, diastolic blood pressure, and heart rate. Continuous (category‐free) NRI and IDI with bootstrap 95% confidence intervals were reported.

Abbreviations: ALI, advanced lung cancer inflammation index; IDI, integrated discriminant improvement; mCONUT, modified Controlling Nutritional Status; NRI, net reclassification index.

To further strengthen the assessment of incremental value, the traditional model was explicitly defined as comprising age, sex, history of diabetes, smoking history, reperfusion time, uric acid, DBP, and heart rate (length of hospital stay excluded). Adding mCONUT to this model increased the optimism‐corrected *C*‐statistic from 0.751 to 0.785, reduced the Brier score from 0.131 to 0.126 (optimism‐corrected 0.133 vs. 0.129), and improved calibration (optimism‐corrected calibration intercept 0.005; calibration slope 0.965; Supporting Information S1: Figure S3). Decision curve analysis showed consistently higher net benefit for the model incorporating mCONUT across threshold probabilities of 5%–40% (Supporting Information S1: Figure S4). The full set of incremental value metrics is summarized in Supporting Information S1: Table S3.

### 3.7 Subgroup Analysis of the Effects of ALI and mCONUT Scores on HF Risk

Subgroup analyses revealed largely consistent effects for both ALI and mCONUT on HF risk across different clinical profiles (Figure 6A,B). While ALI inversely correlated with HF risk in the general cohort, this relationship was only statistically significant among males, non‐smokers, patients of all ages, and those with hypertension but without diabetes (all *p* < 0.05). In contrast, the positive association between higher mCONUT scores and elevated HF risk remained robust and significant across all tested subgroups. We identified only one significant interaction across the analyses: age modified the prognostic impact of mCONUT (interaction *p* = 0.024), yielding a more pronounced effect in patients younger than 60 (OR 1.330) versus those ≥ 60 (OR 1.138). All remaining interaction *p* values exceeded 0.05, highlighting the reliability of mCONUT as an independent risk marker.

**Figure 6:**
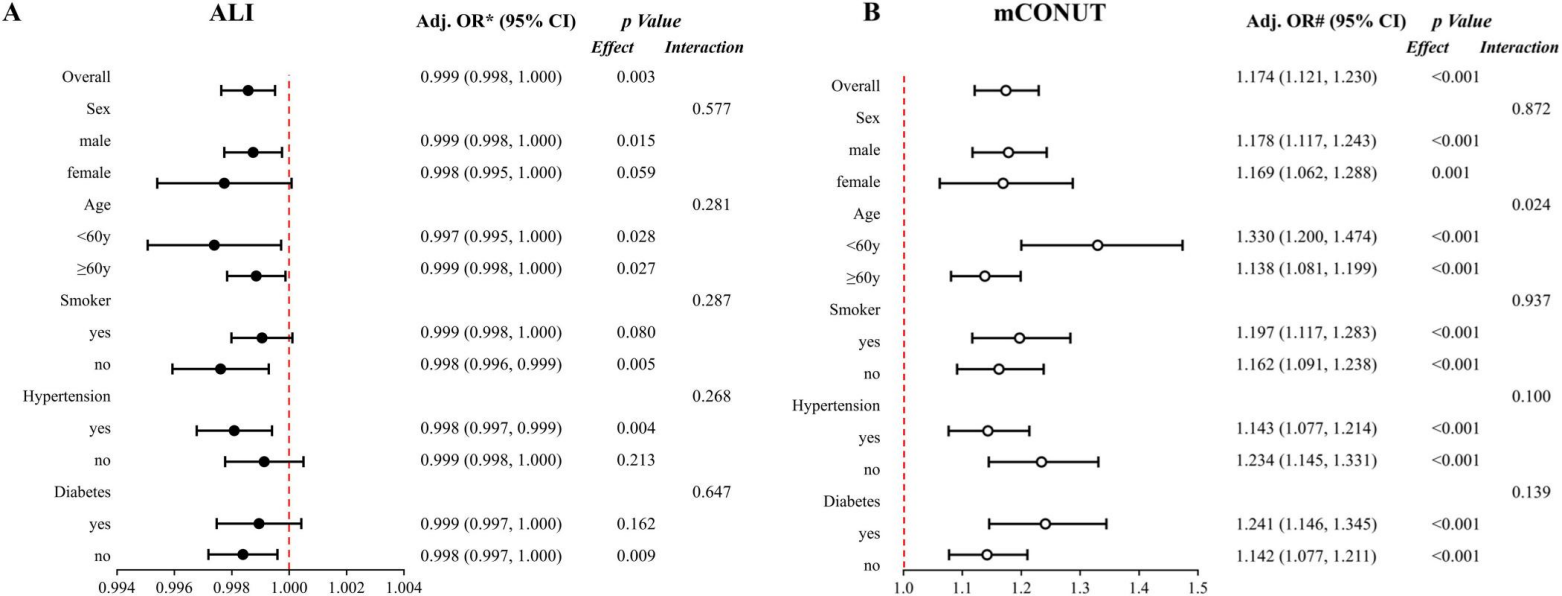
Forest plots showing effects of ALI (A) and mCONUT (B) on de novo heart failure in patients with STEMI subgroup. *Adjusted for BNP, troponin I, LVEF, creatinine, uric acid, CRP, fasting blood glucose, triglycerides, diastolic blood pressure, heart rate, history of diabetes, smoking history, reperfusion time, age, and sex. # Adjusted for BNP, troponin I, LVEF, creatinine, uric acid, fasting blood glucose, triglycerides, diastolic blood pressure, heart rate, history of diabetes, smoking history, reperfusion time, age, sex, and BMI. OR, odds ratio; 95% CI, 95% confidence interval.

### 3.8 Sensitivity Analyses

To verify the independent prognostic value of the composite index after excluding potential collinearity interference (VIF < 5), this sensitivity analysis tested the model by stepwise backfilling of component variables (albumin, BMI, TC, CRP). The results showed that the predictive effect of ALI completely disappeared after adjusting for its core component, albumin (*p* = 0.396), indicating that excluding component variables in the main model was necessary. Conversely, mCONUT remained statistically significant after simultaneously adjusting for all components of albumin, TC, and CRP (*p* = 0.04) (Supporting Information S1: Table S4), strongly demonstrating the rationale for excluding component variables in the main model and confirming the high robustness of mCONUT.

Additional sensitivity analyses further support the robustness of our findings. Adjustment for treatment era, approximated by the admission serial number and modeled both continuously and in quartiles, did not materially change the estimates (ALI OR 0.999; mCONUT OR 1.177; Supporting Information S1: Table S5), and temporal split‐sample validation—developing the model in the early cohort (median year 2018) and validating it in the independent later cohort (2022) preserved the discriminatory increment of mCONUT (AUC improved from 0.754 to 0.793; Supporting Information S1: Table S6). The associations also remained significant after additional adjustment for the Killip class and when restricting the analysis to patients with Killip class I at admission (i.e., without clinical HF at presentation), confirming that the results are independent of admission hemodynamic status (Supporting Information S1: Table S7). Finally, alternative assignments of borderline mCONUT values or alternative CRP cut‐off schemes changed the adjusted ORs by less than 5% (Supporting Information S1: Table S8).

## 4 Discussion

This study focused on first‐time STEMI patients, systematically exploring the value of ALI and mCONUT scores in the occurrence of new‐onset HF during hospitalization. First, this study found significant differences in the clinical characteristics of STEMI patients with new‐onset HF compared to non‐HF patients. This result is consistent with previous studies [20], suggesting that the occurrence of new‐onset HF is closely related to increased myocardial injury, decreased cardiac reserve, and metabolic disorders. Notably, STEMI patients with new‐onset HF had longer hospital stays, higher CRP levels, and lower serum albumin levels, indirectly suggesting that nutritional and inflammatory status may play an important role in the development of new‐onset HF. These findings provide a clinical basis for further exploring the clinical value of ALI and mCONUT scores.

ALI was positively correlated with cardiac function and nutritional indicators (such as EF and triglycerides) and negatively correlated with age and cardiorenal injury indicators (such as BNP, cTNI, and creatinine), suggesting that ALI may serve as a protective indicator of a healthy physiological state. Conversely, the mCONUT score was positively correlated with age, inflammation, and indicators of deteriorating cardiorenal function, and negatively correlated with nutritional status and cardiac function indicators, suggesting that the mCONUT score can effectively reflect malnutrition and systemic inflammatory status.

In STEMI patients, both ALI and mCONUT scores were statistically significantly associated with new‐onset HF. Multivariate regression analysis showed that ALI was independently negatively correlated with the occurrence of HF, and this association persisted after adjusting for traditional risk factors such as BNP, troponin, and EF, suggesting that ALI may affect HF risk through non‐traditional pathways such as systemic inflammation and changes in body composition. Further dose−response analysis showed that the lower the ALI, the higher the short‐term risk of HF. In contrast, the HF group showed higher mCONUT scores and a higher proportion of moderate‐to‐severe malnutrition. Multivariate regression showed that mCONUT was independently positively correlated with the occurrence of HF, and the incidence of HF increased with increasing mCONUT grade. Notably, the mCONUT score demonstrated superior stratification efficacy compared to ALI in risk differentiation. This difference may stem from mCONUT's integration of a more comprehensive pathophysiological dimension, simultaneously reflecting nutritional status (albumin, TC) and immune‐inflammatory status (lymphocyte count). Although the relationship between blood lipids and HF remains controversial [21], higher cholesterol levels in the context of AMI may imply stronger metabolic reserves, aiding in coping with acute stress. The BMI component in ALI is susceptible to interference from acute‐phase fluid retention and edema, and the complex influence of the “obesity paradox” [22] may weaken its robustness in short‐term risk identification. ROC analysis showed that mCONUT's comprehensive reflection of multi‐system states made its discriminative efficacy superior to a single indicator, suggesting that HF is the result of multiple synergistic mechanisms [23]. Although the AUC of ALI combined with mCONUT was slightly higher than that of mCONUT alone, the difference was not statistically significant; sensitivity analysis and NRI/IDI results further confirmed that ALI provided a substantially smaller and clinically less meaningful increment than mCONUT. It is important to emphasize that this study was a retrospective observational design, and the above findings should be considered as exploratory results generating hypotheses, rather than causal inferences. Therefore, mCONUT is more suitable as an auxiliary risk identification marker. Although traditional risk assessment tools [24, 25] have played an important role in risk stratification, their potential clinical value lies in incorporating inflammation and nutritional indicators not previously included, enabling early identification of high‐risk subgroups missed by conventional models and thereby optimizing monitoring and risk assessment strategies.

Consistent with our findings, a recent multicentre study of STEMI patients undergoing primary PCI showed that the ALI was an independent predictor of all‐cause mortality [26]. Moreover, the parent CONUT score has been repeatedly validated in STEMI and acute coronary syndrome populations [16, 27, 28], supporting the applicability of inflammation–nutrition indices to the post‐infarction setting. Importantly, the associations of ALI and mCONUT with de novo HF remained significant after additional adjustment for the Killip class and after restricting the analysis to patients without clinical HF at presentation (Killip class I), indicating that our findings are independent of admission hemodynamic status.

Subgroup analysis showed that mCONUT scores exhibited good consistency and robustness in risk stratification across different clinical subgroups, while ALI may have a more sensitive risk differentiation potential in specific subgroups. The underlying mechanism may be related to differences in inflammatory response, metabolic reserves, and body composition among different populations, which requires further clarification in future studies. Notably, the association of mCONUT with HF was stronger in patients aged < 60 years than in those ≥ 60 years, suggesting that nutritional‐inflammatory status may be particularly informative for risk stratification in younger patients; this observation warrants confirmation in future studies.

Notably, a previous study by our team [29], which partially overlapped with the population in this study, also found a negative correlation between the triglyceride glucose‐BMI index, reflecting insulin resistance, and the risk of HF. These findings, along with the association between ALI, mCONUT, and HF observed in this study, collectively suggest that the synergistic effect of metabolic factors (including blood glucose, triglycerides, and cholesterol) and inflammatory states may be an important pathological basis for the development and progression of HF. Given that the above evidence is based on observational studies, this hypothesis requires further basic and clinical research to verify.

This study found a significant association between ALI and mCONUT scores and new‐onset HF. The underlying biological basis may lie in the malignant synergistic effect between nutritional depletion and systemic inflammation, a process that spans the entire post‐myocardial infarction cycle from acute immune activation to chronic ventricular remodeling. First, during the inflammatory initiation and cytokine storm phase, hypoalbuminemia is not only a marker of nutritional depletion but also reflects a “negative acute response” where the liver preferentially synthesizes pro‐inflammatory proteins such as CRP while inhibiting albumin synthesis [30]. This state is often accompanied by the release of high levels of pro‐inflammatory cytokines (such as IL‐6 and TNF‐α), driving the recruitment of neutrophils and monocytes to the infarcted myocardium [31]. Simultaneously, decreased cholesterol levels not only weaken cell membrane stability and endothelial function, exacerbating microcirculatory disturbances, but may also affect lipid raft‐mediated signal transduction [32], further amplifying oxidative stress. The low lymphocyte count contained in mCONUT indicates a state of lymphatic depletion, leading to a decreased immune surveillance capacity for initial damage and resulting in uncontrolled early inflammatory responses [33]. Second, in the stage of impaired inflammation resolution, effective inflammation resolution depends on the synthesis of specialized pro‐resolution mediators, the precursors of which are polyunsaturated fatty acids and sufficient protein reserves [34]. The nutrient substrate deficiency reflected by ALI and mCONUT may prevent the body from initiating the inflammation resolution process in a timely manner, leading to the continued activation of pro‐inflammatory macrophages (M1 type) and their inability to transform into repair macrophages (M2 type). This failure of inflammation resolution results in the damaged myocardium being chronically exposed to high concentrations of reactive oxygen species and proteases, hindering normal scar formation. Finally, in the stage of adverse ventricular remodeling, persistent chronic low‐grade inflammation promotes excessive fibroblast proliferation and extracellular matrix deposition by activating signaling pathways such as TGF‐β/Smad [35], accelerating the process of myocardial fibrosis. Simultaneously, persistent oxidative stress induces cardiomyocyte apoptosis and hypertrophy, disrupting the integrity of the myocardial contractile units. This pathological cascade driven by nutritional‐immune burden ultimately leads to left ventricular remodeling, decreased compliance, and pump failure.

## 5 Limitations

Several limitations exist in this study. First, as a retrospective single‐center study, true external validation in independent multi‐center cohorts remains a priority. Second, owing to the real‐world workflow of our chest pain center [36], the comprehensive biochemical panels required for ALI and mCONUT calculation were routinely drawn in the fasting state the morning after admission (approximately 12−18 h post‐admission) rather than immediately before primary PCI. While this precludes sensitivity analyses using pre‐PCI values—meaning biomarker levels may partly reflect early reperfusion or fluid resuscitation effects—systemic immunometabolic markers fluctuate far less rapidly than acute necrosis markers, making this next‐morning time point a clinically relevant post‐reperfusion baseline. Third, the lowest LVEF from multiple echocardiograms was used for adjustment to provide a conservative estimate of cardiac reserve. Because the initial post‐PCI echocardiogram was typically obtained within 24−48 h, a window overlapping the peak incidence of de novo HF (median 1.5 days), this value may partly reflect early hemodynamic deterioration in a minority of patients. Furthermore, LVEF values were extracted from routine clinical reports without core‐laboratory reproducibility metrics. Importantly, since LVEF served only as an adjustment covariate, any non‐differential measurement error would tend to attenuate rather than inflate the observed associations, rendering our estimates conservative.

Fourth, the mCONUT score was originally derived in patients with cancer cachexia; thus, its cut‐offs and weighting may require recalibration for cardio‐metabolic populations. Additionally, ALI and mCONUT were not monitored dynamically, and their association with long‐term prognosis requires further exploration. Fifth, despite multivariable adjustment, residual confounding may persist. The extended observation period introduced heterogeneous changes in clinical practice, such as optimized reperfusion times and the transition from ACEI/ARB to ARNI therapies [37]. Although our adjustments for treatment era and temporal split‐sample validation suggested the results are robust to these secular trends, residual bias cannot be fully excluded. Finally, the specific molecular mechanisms by which these inflammatory and metabolic disorders influence HF development were not fully explored. Future prospective multi‐center studies, combined with basic experimental research, are needed to validate the clinical translational value of these findings.

## 6 Conclusion

In summary, this study has demonstrated that ALI and mCONUT are independent predictors of in‐hospital incident HF in STEMI patients. Despite significant progress made in the prevention and treatment of de novo HF following STEMI [38], as a simple and readily available indicator, the mCONUT score can help identify high‐risk patients early and provide new avenues for clinical intervention, such as through nutritional support and anti‐inflammatory therapy, thereby enabling potential improvements in patient outcomes through more personalized management strategies.

## Author Contributions

Zhiyin Dai and Lijuan Yang participated in investigation, data curation, formal analysis, methodology, writing, review, and editing. Rui Chen contributed to conceptualization, investigation, data curation, validation, writing the original draft, writing, review and editing, and funding acquisition. Wei Yuan contributed to supervision, project administration, visualization, writing, review, and editing. Duzhe Jiang and Junjie Liu contributed to data curation, writing, review, and editing. Ye Zhou participated in supervision, project administration, funding acquisition, formal analysis, writing, review, and editing. All authors have made significant contributions to the reported work in one or more of the following aspects: conception, study design, execution, acquisition of data, analysis, and interpretation. They participated in drafting, revising, or critically reviewing the article; approved the final version for publication; agreed on the journal to which the article has been submitted; and agree to be accountable for all aspects of the work. All named authors have read and approved the manuscript for publication.

## Ethics Statement

The study protocol was approved by the Ethics Committee of the Affiliated Hospital of Jiangsu University (Approval No. KY2023K0904). This study was conducted in accordance with the Declaration of Helsinki. The requirement for informed consent was waived by the committee due to the retrospective nature of the study and because all data were derived from standardized examinations performed during routine hospitalization.

## Conflicts of Interest

The authors declare no conflicts of interest.

## Supporting information


Supporting File

## Data Availability

The data that support the findings of this study are available from the corresponding author upon reasonable request.
