# Supplementary material for: Association of ALI and mCONUT With In‐Hospital De Novo Heart Failure in Patients With STEMI
Source: Clin Cardiol. 2026 Nov 11;49(11):e70526. doi: 10.1002/clc.70526 (PMC13753704; doi:10.1002/clc.70526)
Supplement: Supplementary file 1 [file CLC-49-e70526-s001.docx]

**Supplementary Figure S1 Correlation between ALI/ mCONUT and clinical variables.**

BNP: b-type natriuretic peptide, TNI: troponin I, EF: ejection fraction, ALT: alanine aminotransferase, AST: aspartate aminotransferase, Cr: creatinine, UA: uric acid, TG: triglycerides, SBP: systolic blood pressure, DBP: diastolic blood pressure, So-to-B: symptom onset-to- balloon, HR: heart rate, ALI: advanced lung cancer inflammation index, mCONUT: modified Controlling Nutritional Status, CRP: C-reactive protein.

**Supplementary Figure S2 Calibration curves for ALI (A) and mCONUT (B) in predicting post-STEMI heart failure.**

ALI: advanced lung cancer inflammation index, mCONUT: modified Controlling Nutritional Status

**Supplementary Figure S3 Calibration plot of the traditional model and the model incorporating mCONUT for predicting in-hospital de novo HF (deciles of predicted probability).**

Traditional model: age, sex, history of diabetes, smoking history, reperfusion time, uric acid, diastolic blood pressure, and heart rate. Length of hospital stay was excluded.

**Supplementary Figure S4 Decision curve analysis for in-hospital de novo HF.**

The x-axis represents the threshold probability; the y-axis represents the net benefit. Across threshold probabilities of 5%-40%, the model incorporating mCONUT yielded consistently higher net benefit than the traditional model alone and than the treat-all and treat-none strategies.

**Supplementary Table S1 Scoring criteria of the modified Controlling Nutritional Status (mCONUT) score**

| **Parameter (unit)** | **0 points** | **1 / 2 points** | **3 / 4 points** | **5 / 6 points** |
| --- | --- | --- | --- | --- |
| Albumin (g/L) | ≥ 35 (0) | 30–34.9 (2) | 25–29.9 (4) | < 25 (6) |
| Lymphocyte count (×10⁹/L) | ≥ 1.6 (0) | 1.2–1.59 (1) | 0.8–1.19 (2) | < 0.8 (3) |
| Total cholesterol (mmol/L) | ≥ 4.65 (0) | 3.62–4.64 (1) | 2.58–3.61 (2) | < 2.58 (3) |
| CRP (mg/L) [double-weighted] | ≤ 5 (0) | 5.01–10 (2) | 10.01–35 (4) | > 35 (6) |

The total mCONUT score ranges from 0 to 18 points, with higher scores indicating worse nutrition–inflammation status. Borderline rule: a value exactly equal to a cut-off was assigned to the lower-score (normal) category. In the present cohort, the observed score ranged from 0 to 16 (median 3, IQR 2–6).

**Supplementary Table S2** Univariate logistic regression analysis of factors associated with the incidence of *de novo* heart failure in patients with STEMI.

| **Variable** | **OR** | **95% CI** | ***p* value** |
| --- | --- | --- | --- |
| Age | 1.058 | 1.048-1.070 | <0.001 |
| Sex | 2.305 | 1.743-3.049 | <0.001 |
| Current smoker | 1.395 | 1.083-1.798 | 0.01 |
| DM | 1.762 | 1.358-2.285 | <0.001 |
| HTN | 1.104 | 0.852-1.431 | 0.455 |
| BMI | 0.958 | 0.936-0.981 | <0.001 |
| BNP | 1.008 | 1.008-1.009 | <0.001 |
| TNI | 1.019 | 1.007-1.031 | 0.002 |
| LVEF | 0.867 | 0.853-0.882 | <0.001 |
| LOS | 1.110 | 1.078-1.143 | <0.001 |
| ALT | 1.002 | 1.001-1.004 | 0.001 |
| AST | 1.001 | 1.001-1.002 | <0.001 |
| Albumin | 0.830 | 0.798-0.863 | <0.001 |
| Creatinine | 1.010 | 1.007-1.013 | <0.001 |
| Uric acid | 1.004 | 1.003-1.005 | <0.001 |
| FBG | 1.131 | 1.094-1.169 | <0.001 |
| TG | 0.730 | 0.649-0.821 | <0.001 |
| TC | 0.880 | 0.802-0.964 | 0.006 |
| HDL-C | 1.167 | 0.809-1.684 | 0.409 |
| LDL-C | 0.771 | 0.656-0.907 | 0.002 |
| CRP | 1.024 | 1.019-1.030 | <0.001 |
| SBP | 0.994 | 0.987-1.000 | 0.047 |
| DBP | 0.989 | 0.979-0.999 | 0.024 |
| HR | 1.025 | 1.017-1.033 | <0.001 |
| So-to-B | 1.018 | 1.011-1.026 | <0.001 |
| ALI | 0.854 | 0.832-0.876 | <0.001 |
| mCONUT | 1.260 | 1.212-1.311 | <0.001 |

DM: diabetes mellitus, HTN: hypertension, BMI: body mass index, BNP: b-type natriuretic peptide, TNI: troponin I, LVEF: left ventricular ejection fraction, LOS: length of stay, ALT: alanine aminotransferase, AST: aspartate aminotransferase, FBG: fasting blood sugar, TG: triglycerides, TC: total cholesterol, HDL-C: high density lipoprotein cholesterol, LDL-C: low density lipoprotein cholesterol, CRP: C-reactive protein, SBP: systolic blood pressure, DBP: diastolic blood pressure, HR: heart rate, So-to-B: symptom onset-to- balloon, ALI: advanced lung cancer inflammation index, mCONUT: modified Controlling Nutritional Status, OR: odds ratio, 95% CI: 95% confidence interval.

**Supplementary Table S3 Incremental value of adding mCONUT to the traditional model for predicting in-hospital de novo HF**

| **Metric** | **Traditional model** | **Traditional model + mCONUT** | **Difference / Comment** |
| --- | --- | --- | --- |
| Apparent AUC (C-statistic) | 0.758 | 0.792 | ΔAUC = 0.034 (bootstrap 95% CI 0.016–0.052) |
| Optimism-corrected AUC (400 bootstrap resamples) | 0.751 | 0.785 | Increment preserved; minimal overfitting |
| Brier score (apparent) | 0.1311 | 0.1262 | Absolute improvement 0.0049 |
| Brier score (optimism-corrected) | 0.1334 | 0.1287 | Improvement preserved |
| Calibration-in-the-large (optimism-corrected) | 0.005 | 0.005 | Both values approach 0 (well calibrated) |
| Calibration slope (optimism-corrected) | 0.965 | 0.965 | Both values approach 1 (well calibrated) |
| Continuous NRI (95% CI) | — | 0.444 (0.313–0.566) | *p* < 0.001 |
| IDI (95% CI) | — | 0.034 (0.021–0.047) | *p* < 0.001 |

Traditional model: age, sex, history of diabetes, smoking history, reperfusion time, uric acid, diastolic blood pressure, and heart rate. Length of hospital stay was excluded. Optimism correction was performed with 400 bootstrap resamples. Corresponding figures: Supplementary Figures S3 and S4.

**Supplementary Table S4 Sensitivity analysis of models based on ALI and mCONUT**

|  | **OR** | **95% CI** | ***p* value** |
| --- | --- | --- | --- |
| ALI |  |  |  |
| Model 2 | 0.999 | 0.998-1.000 | 0.003 |
| Model 2+ albumin | 0.997 | 0.987-1.005 | 0.396 |
| Model 2+ albumin+BMI | 0.997 | 0.986-1.005 | 0.395 |
|  |  |  |  |
| mCONUT |  |  |  |
| Model 3 | 1.174 | 1.121-1.230 | < 0.001 |
| Model 3+ albumin | 1.167 | 1.090-1.242 | < 0.001 |
| Model 3+ albumin+TC | 1.167 | 1.086-1.247 | < 0.001 |
| Model 3+ albumin+TC+CRP | 1.092 | 1-1.186 | 0.04 |

Model 2: BNP, troponin I, LVEF, creatinine, uric acid, CRP, fasting blood glucose, triglycerides, diastolic blood pressure, heart rate, history of diabetes, smoking history, reperfusion time, age, and sex.

Model 3: BNP, troponin I, LVEF, creatinine, uric acid, fasting blood glucose, triglyceride, diastolic blood pressure, heart rate, history of diabetes, smoking history, reperfusion time, age, sex, and BMI.

BMI: body mass index, CRP: C-reactive protein, TC: total cholesterol, ALI: advanced lung cancer inflammation index, mCONUT: modified Controlling Nutritional Status, OR: odds ratio, 95% CI: 95% confidence interval.

**Supplementary Table S5 Effect estimates before and after adjustment for treatment era**

| **Exposure / Model** | **Without era adjustment** | **Adjusted for era (quartiles)** | **Adjusted for era (continuous)** |
| --- | --- | --- | --- |
| ALI - Model 2 | 0.999 (0.998, 1.000) , *p* = 0.003 | 0.999 (0.998, 1.000),  *p* = 0.003 | 0.999 (0.998, 1.000),  *p* = 0.002 |
| mCONUT - Model 3 | 1.174 (1.121, 1.230),  *p* < 0.001 | 1.177 (1.123, 1.233) ,  *p* < 0.001 | 1.176 (1.122, 1.231),  *p* < 0.001 |

The treatment era was approximated by the hospital admission serial number (a monotonically increasing calendar-time proxy covering 2016–2025) and modeled both as a continuous year-equivalent and in quartiles.

**Supplementary Table S6 Temporal split-sample validation**

| **Cohort** | **Traditional model AUC** | **Traditional + mCONUT AUC** | **ΔAUC** |
| --- | --- | --- | --- |
| Early cohort (development; median year 2018, n = 800) | 0.756 | 0.777 | 0.021 |
| Later cohort (independent validation; median year 2022, n = 798) | 0.754 | 0.793 | 0.039 |

The cohort was divided according to the median hospital admission serial number. The model was developed on the early cohort and evaluated on the independent later cohort; the discriminatory increment of mCONUT was preserved.

**Supplementary Table S7 Sensitivity analyses regarding the Killip class**

| **Model** | **Main analysis (no Killip)** | **Sensitivity A: + Killip class** | **Sensitivity B: Killip I only** |
| --- | --- | --- | --- |
| ALI — Model 2,  OR (95% CI) | 0.999 (0.998, 1.000) | 0.999 (0.998, 1.000) | 0.999 (0.998, 1.000) |
| *p* value | 0.003 | 0.011 | 0.029 |
| mCONUT —  Model 3, OR (95% CI) | 1.174 (1.121, 1.230) | 1.168 (1.114, 1.225) | 1.169 (1.106, 1.236) |
| *p* value | <0.001 | <0.001 | <0.001 |

Sensitivity A: Killip II–III and Killip IV entered as dummy variables with Killip I as reference. Sensitivity B: analysis restricted to patients with Killip class I at admission.

Model 2: Adjusted for BNP, troponin I, LVEF, creatinine, uric acid, CRP, fasting blood glucose, triglycerides, diastolic blood pressure, heart rate, history of diabetes, smoking history, reperfusion time, age, and sex.

Model 3: Adjusted for BNP, troponin I, LVEF, creatinine, uric acid, fasting blood glucose, triglyceride, diastolic blood pressure, heart rate, history of diabetes, smoking history, reperfusion time, age, sex, and BMI.

**Supplementary Table S8 Sensitivity analyses of the mCONUT scoring scheme**

**Panel A. Alternative assignments of borderline values**

| **Scoring scheme** | **Unadjusted OR (95% CI)** | **Adjusted OR (95% CI)*** |
| --- | --- | --- |
| Primary analysis (Zhang 2024 scheme) | 1.260 (1.212–1.311) | 1.174 (1.121–1.230) |
| CRP = 5.0 mg/L scored 2 points | 1.265 (1.216–1.317) | 1.181 (1.127–1.238) |
| Lymphocyte 1.6×10⁹/L scored 1 point | 1.268 (1.218–1.319) | 1.183 (1.129–1.241) |
| Total cholesterol 4.65 mmol/L scored 1 point | 1.266 (1.217–1.318) | 1.183 (1.128–1.240) |
| Albumin 35 g/L scored 2 points | 1.267 (1.218–1.319) | 1.182 (1.128–1.239) |

**Panel B. Alternative CRP cut-off schemes**

| **CRP scoring scheme** | **Unadjusted OR (95% CI)** | **Adjusted OR (95% CI)*** |
| --- | --- | --- |
| Zhang 2024 cut-offs (≤5 / ≤10 / ≤35 mg/L) | 1.260 (1.212–1.311) | 1.174 (1.121–1.230) |
| Alternative cut-offs (<10 / ≤35 / >35 mg/L) | 1.260 (1.210–1.313) | 1.179 (1.123–1.237) |
| Without CRP (i.e., the original CONUT score) | 1.262 (1.187–1.341) | 1.160 (1.077–1.249) |

* Adjusted for BNP, troponin I, LVEF, creatinine, uric acid, fasting blood glucose, triglyceride, diastolic blood pressure, heart rate, history of diabetes, smoking history, reperfusion time, age, sex, and BMI. All variants changed the ORs by less than 5% and did not alter any conclusion. CRP was measured and expressed in mg/L.
